# Lenz microphthalmia syndrome in neurosurgical practice: a case report and review of the literature

**DOI:** 10.1007/s00381-020-05035-1

**Published:** 2021-01-25

**Authors:** Matteo Monticelli, Raffaele De Marco, Diego Garbossa

**Affiliations:** Neurosurgery Unit, Department of Neuroscience “Rita Levi Montalcini”, “Città della Salute e della Scienza” University Hospital, Turin University, Via Cherasco, 15, 10126 Turin, Italy

**Keywords:** Lenz microphthalmia syndrome, Traumatic brain injury, Traumatic intracerebral hemorrhage, Decompressive craniectomy

## Abstract

Lenz microphthalmia syndrome (LMS) is an allelic X-linked syndrome correlated to a null mutation of B cell lymphoma (BCL-6) corepressor (BCOR) gene, which is essential in the early embryonic development. Phenotypically, this rare hereditary syndrome is characterized by microphthalmia/anophthalmia and other eye disorders; mental disability; dental, ear, and digital abnormalities; and variable malformations affecting the heart, skeleton (limbs and/or spine), and genitourinary tract. In this paper, a case of a young adult with LMS affected additionally by immuno-hematological disturbances was treated with decompressive craniectomy after domestic accidental fall. Case description and a brief review of the current literature about this rare condition are presented here.

## Introduction

Lenz microphthalmia syndrome (LMS) is a rare genetic disorder, inherited in an X-linked fashion. Since it first appeared in the scientific landscape, LMS was considered as an allelic variant of the oculofaciocardiodental (OFCD) syndrome, another and better-understood X-linked microphthalmia syndrome, also known as MCOSP2, caused by a mutation in the B cell lymphoma (BCL-6) corepressor (BCOR) gene. It is not the aim of this paper to deepen the molecular aspect of this condition but, generically speaking, the product the BCOR gene seems to be essential during the early process of embryogenesis, particularly in the development of the eye and, variably, in other organs as well. There are only few papers in literature regarding this topic, mostly case reports. The first widest case series, at the authors best knowledge, was reviewed considering just twelve patients as reported by Traboulsi et al. in 1988 [1–11], underlying the extreme infrequency with those patients came across neurosurgical everyday clinical practice. Since then, other few cases have been reported, but none of them got involved with the neurosurgical field [12–21].

## Materials and methods

We performed a MEDLINE and PUBMED review of the medical literature using as keywords “Lenz microphthalmia syndrome”, “traumatic brain injury”, “traumatic intracerebral hemorrhage”, and “decompressive craniectomy”. Only articles in English were considered.

The results are summarized in Table 1.

**Table 1:** Review of LMS clinical manifestations in the literature

References	Cataract	Coloboma	Ear anomalies	Palate and teeth anomalies	Microcephaly	Mental retardation	Limb defects	Cardiac anomalies	Genitourinary tract anomalies
Lenz, 1955 (4) [1]	+	−	−	+	−	NR	+	+	+
Hoefnagel et al., 1963 (4) [2]	NR	−	NR	NR	NR	NR	+	NR	+
Hermann and Opitz 1969 (1) [4]	−	+	+	+	+	+	+	NR	+
Goldberg and McKusick, 1971 (4) [5]	+	+	+	+	+	+	+	−	−
Ogunye et al., 1975 (3) [6]	+	−	+	−	+	+	+	NR	+
Dinno et al., 1976 (5) [7]	NR	−	+	−	+	+	+	NR	+
Baraitser et al., 1982 (1) [8]	−	−	+	+	+	+	NR	−	−
Glanz et al., 1983 (1) [9]	NR	−	+	+	+	+	+	NR	+
Pallotta 1983 (1) [10]	−	+	+	+	+	+	+	+	+
Brunquell et al., 1984 (1)* [11]	−	−	+	+	+	+	−	−	+
Traboulsi et al., 1988 (2) [3]	−	+	+	+	+	+	+	+	+
Graham et al., 1991 (4) [12]	NR	−	+	+	NR	+	−	NR	−
Antoniades et al., 1993 (1) [13]	−	+	+	+	+	+	+	NR	−
Ozkinay et al., 1997 (1) [15]	NR	−	+	+	+	+	+	NR	+
Temtamy et al., 2000 (3) [16]	NR	+	+	+	+ [1]	+	+	NR	−
Forrester et al., 2001 (4) [17]	NR	−	+	+	+	+	+	+	+
Gupta et al., 2007 (1) [19]	NR	NR	+	NR	+	ND	+	−	+
Okumus et al., 2008 (1) [20]	NR	NR	+	+	NR	ND	+	+	+
Derman et al., 2011 (1) [21]	NR	NR	NR	+	+	+	+	−	+
Sohil et al. 2013 (1) [22]	−	−	+	+	ND	ND	+	NR	+

+, present; −, absent; *NR*, not reported; *ND*, not yet defined; in brackets are the specified numbers of patients analyzed by those authors

*It follows one of the four cases reported by Hoefnagel et al. in 1963

## Case presentation

The subject was a 28-year-old male (height: 153 cm; weight: 46 kg at the evaluation) affected with Lenz microphthalmia syndrome diagnosed when the patient was 3 years old because of the presence of some typical phenotypic aspects of the syndrome as well: he presented right microphthalmia with iris, optic nerve, and choroid coloboma; bilateral V finger clinodactyly and hammer toe deformity; high arched palate; cryptorchidism (surgically treated after hormonal replacement); and mild aortic insufficiency with an ejection fraction (EF) of 50%, without any sign of heart failure and mental retardation.

Since 1999, the patient was in hematological follow-up for hemolytic autoimmune anemia, thrombocytopenia, and mild neutropenia, defining a picture of autoimmune lymphoproliferative (ALP)–like syndrome. The hematological follow-up and immunological follow-up were characterized by several episodes of hypogammaglobulinemia that have been treated by cyclic administration of intravenous IgG and, more recently, due to the recurrence of autoimmune thrombocytopenia with several hemorrhagic manifestations, by weekly administration of vitamin K.

In 1999, as a result of the chronic therapy with steroids, the patient developed a cerebral aspergilloma which was surgically treated without leaving to him any additional neurological deficit but, after that, he developed residual seizures which were treated with chronic administration of antiepileptic drugs (lacosamide 300 mg × 2/die).

On May 2018, the patient was admitted to the emergency room (ER) of “Città della Salute e della Scienza” Trauma Center in Turin, Italy, after a mild traumatic brain injury which occurred at home about 2 h before.

His usual domestic therapy consisted of mycophenolate mofetil, intravenous immunoglobulin (IVIg) (1 infusion/2 weeks), testosterone, phytomenadione (vitamin K), dorzolamyd + timolol collyrium, calcium phosphate, and lacosamide.

**Fig. 1:**
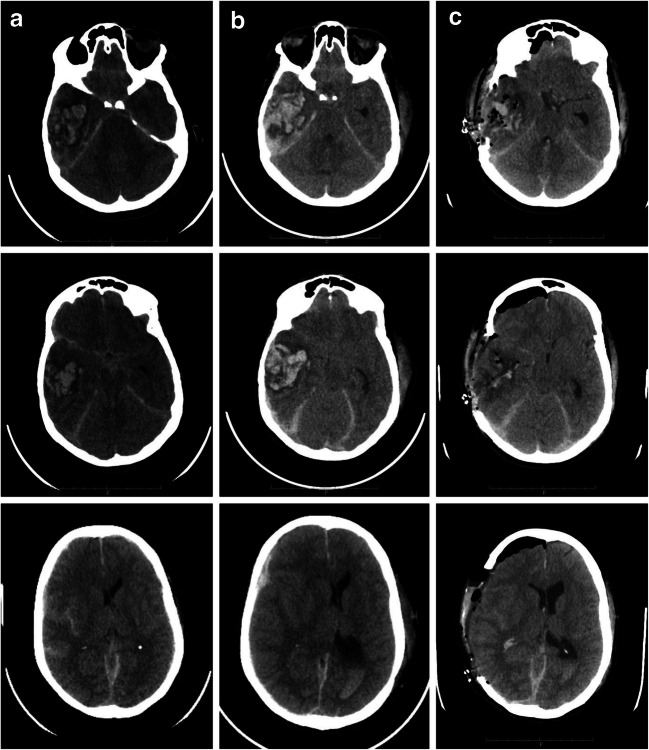
CT scan brain trauma survey for traumatic brain injury in LMS case. **a** From top to bottom, different slices of CT scan at the admission to ED department. **b** Same slices from top to bottom, registered at 6 h from the first CT scan; it could be noted an increase and a better consolidation of the intraparenchymal hematoma in the right temporal lobe; furthermore, an intraventricular hemorrhage and a worsening of the midline shift toward the left hemisphere are shown. **c** Postoperative CT scan; the different slices show the result of craniectomy and right temporal ICH evacuation, with significant reduction of midline shift and an initial reappearance of basal cisterns

The Glasgow Coma Scale (GCS) at the admission slightly oscillates from 15 to 14/15 (M6 V5 E3) without showing any neurological focal deficit. A baseline CT scan was performed (Fig. 1a), showing the presence of a right temporal intracerebral hemorrhage (ICH) with initial mass effect and midline shift. It was remarkable the fact that the ICH was contralateral to the direct traumatic area, so developed as countercoup injury.

Complete blood count (CBC) at ER admission showed 135,000/mmc platelets (PLTS) with an INR ratio of 1.24 (0.8–1.2), and APTT was 30.5 s (25–38) with a ratio of 0.97 (0.8–1.18), white blood cells (WBC) 7790/mmc, and neutrophils 58.5%.

Due to his good neurological status at the examination, a “watch and wait” approach was chosen, meanwhile a prophylactic steroid therapy (dexamethasone 4 mg × 2/day) was started, and a new CT scan follow-up after 6 h from the first one was programmed according to the “Città della Salute e della Scienza” University Hospital guidelines.

This last exam (CT scan after 6 h from the first one) showed a significant increase of the temporal hematoma and consequentially an increased mass effect, an increased midline shift, the near-total obliteration of basal cisterns, and initial radiological signs of a transtentorial herniation as well (Fig. 1b). Clinically speaking, the patient appearance was less responsive to stimuli and less contactable, and he also was developing motor deficits on his left upper and lower arm. At that point, surgery was undoubted.

Under general anesthesia, a standard right decompressive hemicraniectomy with partial hematoma evacuation was performed (Fig. 1c).

The surgical treatment was uneventful and executed without intraoperative complications. However, when the surgery ended, before the anesthesiologist started awakening procedures, the electrocardiogram of the patient suddenly reported ventricular fibrillation. Immediately, cardiopulmonary resuscitation (CPR) procedures started following the Advanced Life Support (ALS) guidelines: an electrical early defibrillation, which was performed at least for five times, and one administration of intravenous epinephrine was necessary to obtain return of spontaneous circulation (ROSC) after a period of 30 min.

An arterial blood gas test performed during the previous maneuvers showed inexplicably a loss of eight points of hemoglobin, from 15.1 g/dL preoperatively to 7.4 g/dL. For this reason, four bags of packed blood cells were performed showing an increase of hemoglobin of 2.4 points (final value 9.8 g/dL). Once the patient’s vital parameters became stable, he was transferred to the intensive care unit (ICU).

During patient’s stay in the ICU, it was assisted another episode of cardiocirculatory arrest, during a first attempt of sedation reduction and extubating maneuvers. Another CPR was performed, obtaining an efficacious recovery of cardiac rhythm and circulatory signs. A transthoracic echocardiography showed severe worsening of the cardiac performance (EF 30%) in a context of apical akinesia, medial segment hypokinesia, and apical hypertrabeculation of the ventricles.

Nevertheless, the cardiological follow-up showed a better cardiac performance (EF 45–50% in NYHA I). It is worth noting to highlight the absence of any clinically significant cardiac malformation before surgery, as demonstrated by serial cardiological examinations done in the previous years.

**Fig. 2:**
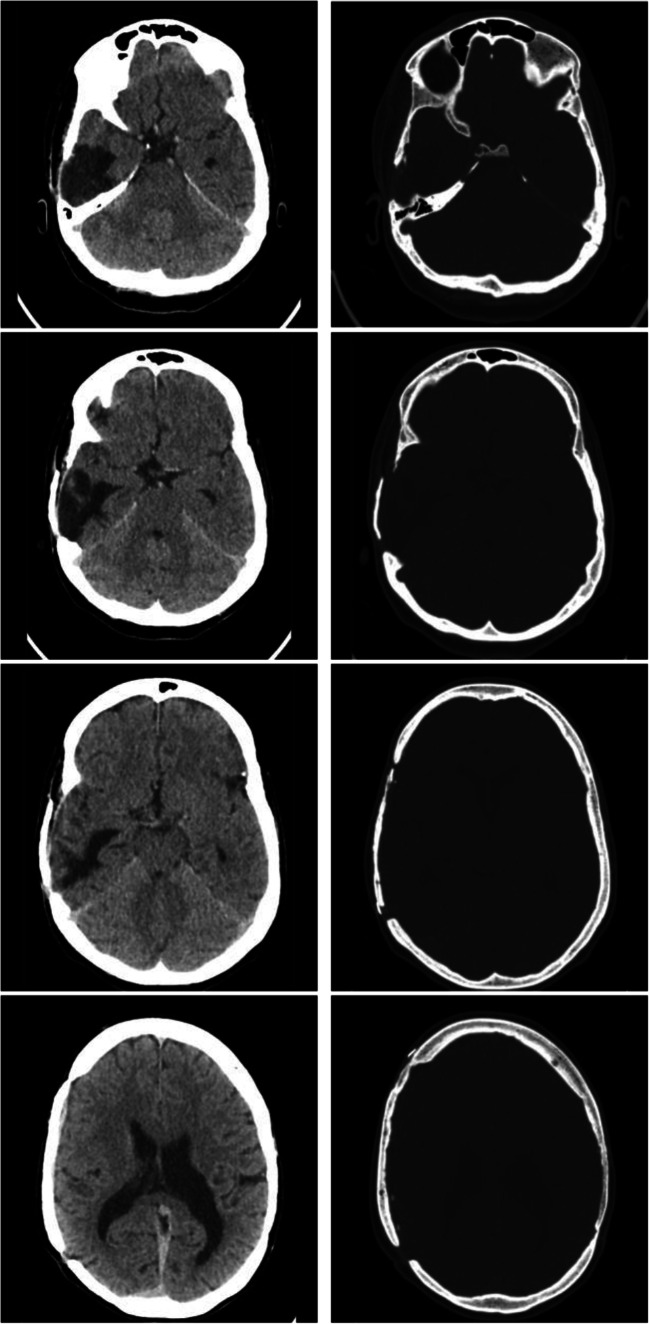
1-year radiological follow-up. The CT scan performed at 1 year from autologous bone flap replacement with different CT windows. On the left, the brain window shows the normal evolution of the right temporal intraparenchymal hemorrhage, with a better definition of a porencephalic area and resolution of any hyperdensity. On the right, the bone window highlights the autologous cranioplasty fixated with titanium plaques and screws to the calvaria

The remaining stay was uneventful and on the 25th postoperative day, the patient was discharged from the hospital and he was sent to a rehabilitation structure to complete the healing. An autologous cranioplasty was made on 31 August 2018 without any problem and the patient was restored to his normal life. At a 1-year follow-up visit, neither neurological deficits nor complications related to the cranioplasty were reported (Fig. 2).

## Discussion

Lenz microphthalmia syndrome is a genetic disorder inherited in an X-linked recessive pattern. Few cases have been reported in the literature since the first report by Lenz et al. in the 1955. Recently, more information have been obtained about the gene involved in these types of syndromic conditions: mutations, such as deletions or insertions/deletions with frameshift in BCL-6-interacting repressor (BCOR) gene, are usually cause by a spectrum of syndrome among which there is LMS. Clinical and linkage analyses suggest that it may be etiologically heterogeneous, linked to both Xq27-q281 (MCOPS1; MIM 309800) and Xp11.42,3 (MCOPS2; MIM 300166) [17, 23, 24].

Specifically, the MCOPS2 form of Lenz microphthalmia syndrome has been shown to be caused by mutation of the BCL-6 corepressor gene (BCOR), while the genetic etiology of MCOPS1, otherwise commonly called Lenz microphthalmia syndrome, remains unknown [25]. Until now, eleven syndromic conditions characterized by microphthalmia have been connected to a mutation in the X chromosome; clearly, there is a clinical overlap between these syndromes.

The typical phenotype of a patient affected by this genetic disorder comprehends a wide range of eye disorders: micro- or anophthalmia is the most frequent; other defects, which are often seen, are coloboma, congenital cataracts, nystagmus, and high incidence of glaucoma.

Among the extraocular anomalies, various degrees of mental retardation, palatal and dental anomalies, congenital heart defects (atrial/ventricular septal defects, other complex heart defects), skeletal defects (such as clinodactyly, syndactyly, or radioulnar synostosis), unilateral renal aplasia, and cryptorchidism are reported. The cardiac defects are the most variable among all the features of this syndrome, as evidenced in the literature (see Table 1). The prominent feature is an innocent cardiac murmur or a mild valve defect; our patient confirms this trend, at least until the sudden worsening after anesthesiologic awakening, when the condition fell down for an extremely rapid decrease of hemoglobin, likely connected to the ALP-like syndrome.

There is no mention in the literature of LMS case with hematological and/or immunological involvement. Abnormal behavior of the immune system and/or impairment of CBC are sometimes linked to in genes located to the X chromosome, like immunodysregulation polyendocrinopathy enteropathy X-linked (IPEX) syndrome, linked to the dysfunction of the transcription factor FOXP3, or Wiskott-Aldrich syndrome, another X-linked condition, caused by a mutation in the gene WAS, located on the short arm of X chromosome (Xp11.23); the latter is not so far from the one hypothesized for MCOPS1.

## Conclusion

To the best of our knowledge, the patient is the first report in the literature of hematologic and immunologic impairment in the setting of this hereditary condition. The decreased number and, probably, the impaired function of the platelets promoted the unusual evolution of a mild traumatic injury considering the patient age and the low-energy dynamic of the brain trauma. Maybe the reactivation of antibodies against the blood cells could explain the sudden and sharp decrease of the hemoglobin in the operative room, causing a cardiac dysfunction, although the EF was near-normal before the surgical procedure.
